# Open vs Percutaneous Moberg Osteotomy With Dorsal Cheilectomy for Hallux Rigidus: A Retrospective Comparative Study of Surgical Approaches With Minimum 2 Years’ Follow-up

**DOI:** 10.1177/24730114251388095

**Published:** 2025-12-10

**Authors:** Alice Montagna, Paolo Ivan Fiore, Enrico Pozzessere, Ettore Vulcano

**Affiliations:** 1 Clinica Ortopedica e Traumatologica, Fondazione IRCCS Policlinico San Matteo, Pavia, Italy; 2 Service of Orthopaedics and Traumatology, Department of Surgery, EOC, Lugano, Switzerland; 3 Foot and Ankle Division of Orthpaedics, Duke University School of Medicine, Durham, NC, USA; 4 Columbia University Division of Orthopedics at Mount Sinai Medical Center, Miami Beach, FL, USA

**Keywords:** Moberg osteotomy, cheilectomy, hallux rigidus, percutaneous surgery, minimally invasive surgery, joint sparing surgery

## Abstract

**Background:**

Hallux rigidus is a degenerative condition affecting the first metatarsophalangeal (MTP) joint, characterized by pain and limited dorsiflexion. In early stages (grades I and II), joint-preserving procedures such as dorsal cheilectomy and Moberg osteotomy are commonly employed. Although traditionally performed through an open approach, the Moberg osteotomy has been adapted to percutaneous techniques, which may offer advantages including reduced soft tissue trauma and faster recovery. This study compares clinical outcomes, postoperative pain, and complication rates between open and percutaneous Moberg osteotomy, both combined with dorsal cheilectomy, in patients with early-stage hallux rigidus.

**Methods:**

A retrospective analysis was conducted on 96 patients who underwent either open (n = 43) or percutaneous (n = 53) Moberg osteotomy in combination with dorsal cheilectomy. All had failed at least 3 months of nonoperative treatment. Exclusion criteria included prior first-ray surgery and advanced hallux rigidus (grades III and IV). Outcomes included the visual analog scale (VAS), Foot Function Index (FFI), and the number of oxycodone tablets consumed during the first 2 postoperative weeks. Complications and reoperations were also documented.

**Results:**

Both groups showed significant improvements in VAS and FFI scores at a minimum of 24-month follow-up, with no statistically significant differences between them. However, postoperative opioid consumption was significantly lower in the percutaneous group compared with the open group (3.6 ± 1.9 vs 13.3 ± 6.1 tablets over 2 weeks; *P* < .0001).Wound complications occurred in 4.7% of the open group and 0% of the percutaneous group (*P* = .20). Reoperation rates were comparable, with 2.3% in the open group and 1.9% in the percutaneous group.

**Conclusion:**

Percutaneous Moberg osteotomy with dorsal cheilectomy is a safe and effective treatment for early-stage hallux rigidus, yielding comparable functional and pain outcomes to the open technique, with lower early postoperative opioid use in this cohort.

**Level of Evidence:**

Level III, retrospective comparative study.

## Introduction

Hallux rigidus is a degenerative condition affecting the first metatarsophalangeal (MTP) joint, characterized by pain, stiffness, and progressive loss of dorsiflexion.^1,2^ It is a common form of arthritis in the foot, particularly affecting adults aged ≥50 years, with a prevalence of 20% to 30% in this age group.^3^ According to the Coughlin and Shurnas classification, the condition is graded from I to IV based on clinical symptoms, radiographic findings, and range of motion.^4,5^ In the early stages (grades I and II), where joint space is preserved and osteophyte formation is moderate, joint-preserving procedures are typically preferred.^6,7^

**Figure 1.**
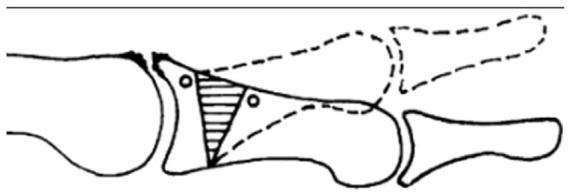
Schematic representation of the Moberg osteotomy (adapted from Issa et al).^14^ The image shows a simplified diagram of a dorsal closing wedge osteotomy of the proximal phalanx of the hallux.

Among these options, the Moberg osteotomy has gained popularity due to its biomechanical advantages and favorable clinical outcomes.^8,9^ This procedure involves a dorsal closing wedge osteotomy of the proximal phalanx, which shifts joint contact pressure plantarly, enhancing dorsiflexion and improving gait mechanics^10,11^ (Figure 1). It is commonly performed in combination with dorsal cheilectomy, which involves resecting dorsal osteophytes and improving joint mobility.^12^ The combination of cheilectomy and Moberg osteotomy is generally indicated for patients with grade I or II hallux rigidus, where preservation of the joint surface remains a viable goal.^13^

Traditionally, the Moberg osteotomy has been performed through an open approach.^14^ However, advancements in minimally invasive techniques have led to the development of percutaneous methods, which aim to minimize soft tissue trauma, reduce postoperative pain, and shorten recovery time. Despite the increasing interest in percutaneous Moberg osteotomy, comparative clinical data with the open technique remain limited.^15^

The present study aims at comparing clinical outcomes, analgesic consumption, and complication rates between open and percutaneous Moberg osteotomy, both performed in conjunction with dorsal cheilectomy, in patients with grade I or II hallux rigidus. By evaluating pain scores, functional outcomes, and postoperative opioid use, we hypothesize that the percutaneous technique is a safe and effective procedure relative to the traditional open approach.

## Materials and Methods

This retrospective study compared 2 surgical techniques for Moberg osteotomy in the treatment of hallux rigidus: an open approach and a percutaneous approach. The Moberg osteotomy is always performed in association with cheilectomy in order to increase dorsiflexion of the proximal phalanx, thereby improving the efficiency of the third rocker phase of gait.

The open group included 43 consecutive patients who underwent surgery between September 2016 and October 2018, whereas the percutaneous group included 53 consecutive patients treated between October 2018 and February 2022.

Inclusion criteria were adult patients undergoing isolated Moberg osteotomy with dorsal cheilectomy for grade I or II hallux rigidus with persistent symptoms despite at least 3 months of footwear modifications and nonoperative treatment, and availability of complete clinical follow-up. Exclusion criteria included revision procedures, grade III or IV hallux rigidus, positive grind test, and any additional surgical interventions on the first ray, such as Lapidus arthrodesis, Keller resection, or first MTP joint fusion.

Postoperative management was identical for both groups: all patients were allowed immediate full weightbearing using a flat postoperative shoe for 6 weeks. After this period, they transitioned to regular footwear as tolerated.

For each patient, data were collected both preoperatively and postoperatively at last follow-up using the visual analog scale (VAS) and the Foot Function Index (FFI).^16^

The FFI is a tool used to assess foot-related disability, pain, and function. It consists of 3 main components: pain, disability, activity limitation. The total FFI score is calculated by summing the scores of all 3 components, with higher scores indicating greater disability and lower function.^17^ Although it was not developed specifically for hallux rigidus, its domains capture key aspects of pain and function that are directly relevant to this pathology.

The number of oxycodone tablets consumed in the first 2 postoperative weeks, complications, and reoperation rates were also recorded.

### Surgical Procedure

#### Open technique

After performing a popliteal nerve block, a dorsal incision was centered over the hallux MTP to expose the joint. With the use of an oscillating saw, a cheilectomy was performed to remove about 20% of the dorsal metatarsal head. Next, an oscillating saw was used to remove a 3-mm dorsal closing wedge at the base of the proximal phalanx. The osteotomy was then manually reduced and stabilized with a 3-mm headless screw.

#### MIS technique

**Figure 2.**
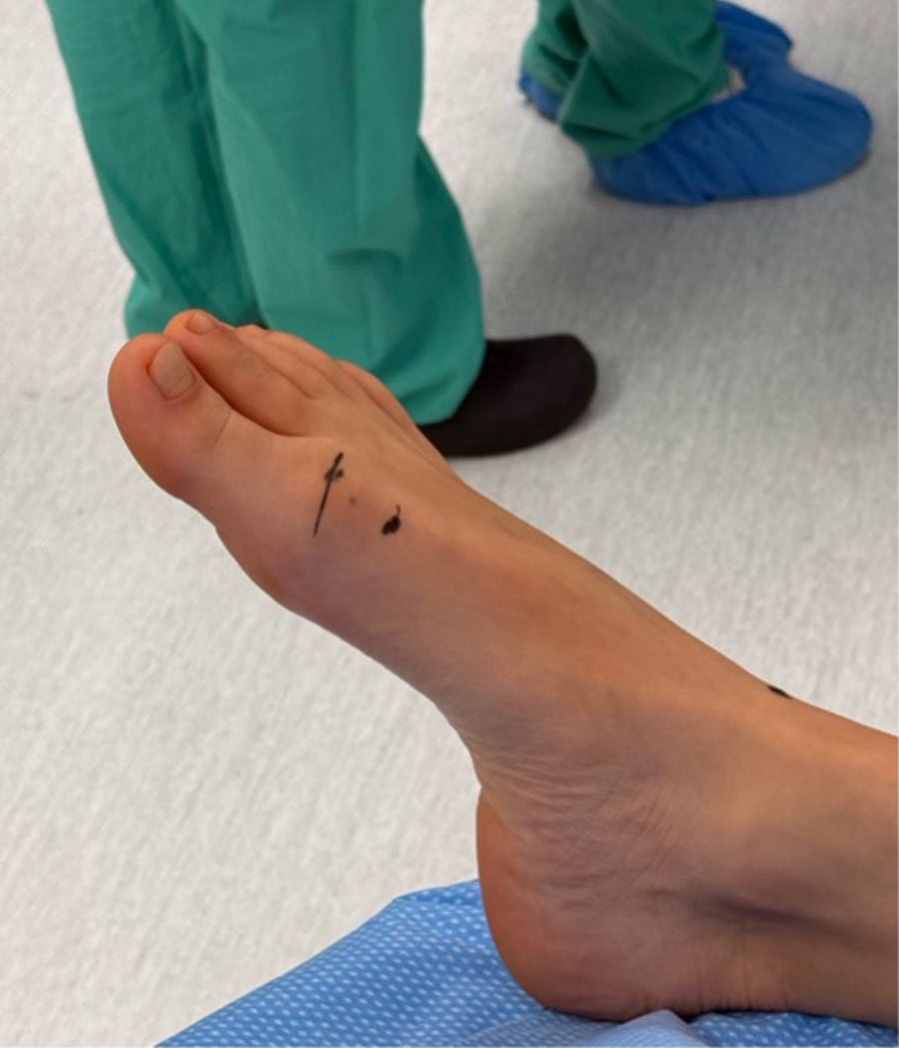
Anatomical landmark for cheilectomy incision. The image shows the cutaneous reference point for the surgical approach, located just medial to the extensor hallucis longus tendon, approximately 2 cm proximal to the first metatarsophalangeal (MTP) joint.

**Figure 3.**
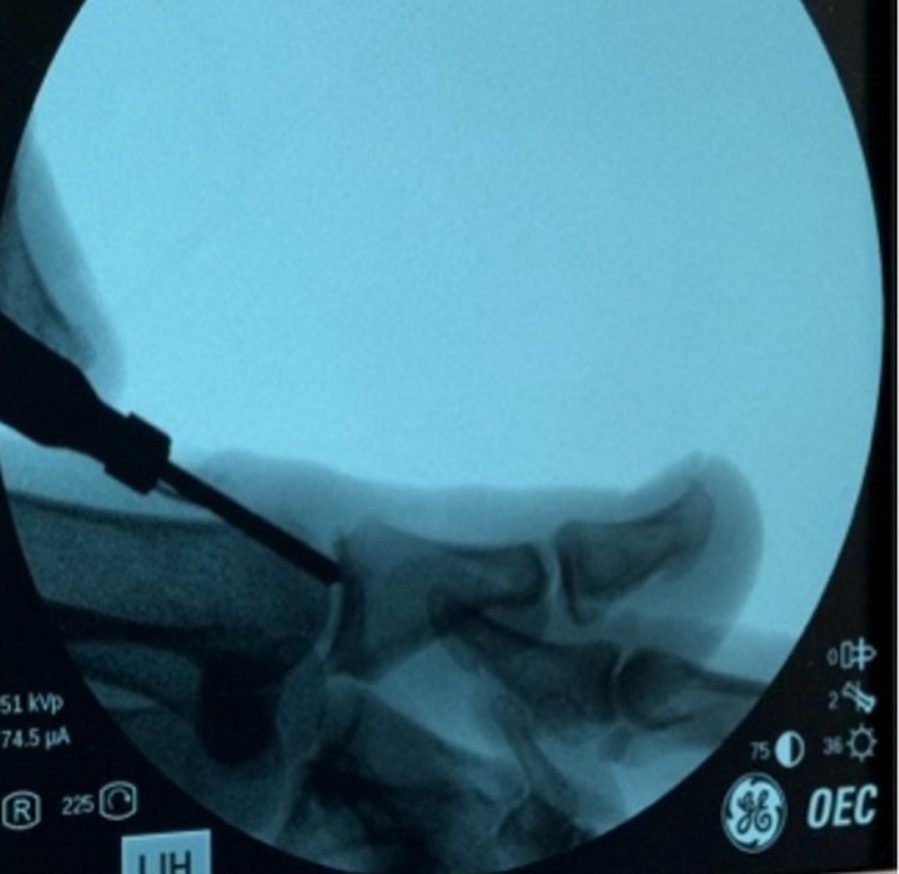
Intraoperative fluoroscopic images (mini C-arm) of the first ray during cheilectomy. The panel shows fluoroscopic guidance during dorsal cheilectomy of the first metatarsal head.

**Figure 4.**
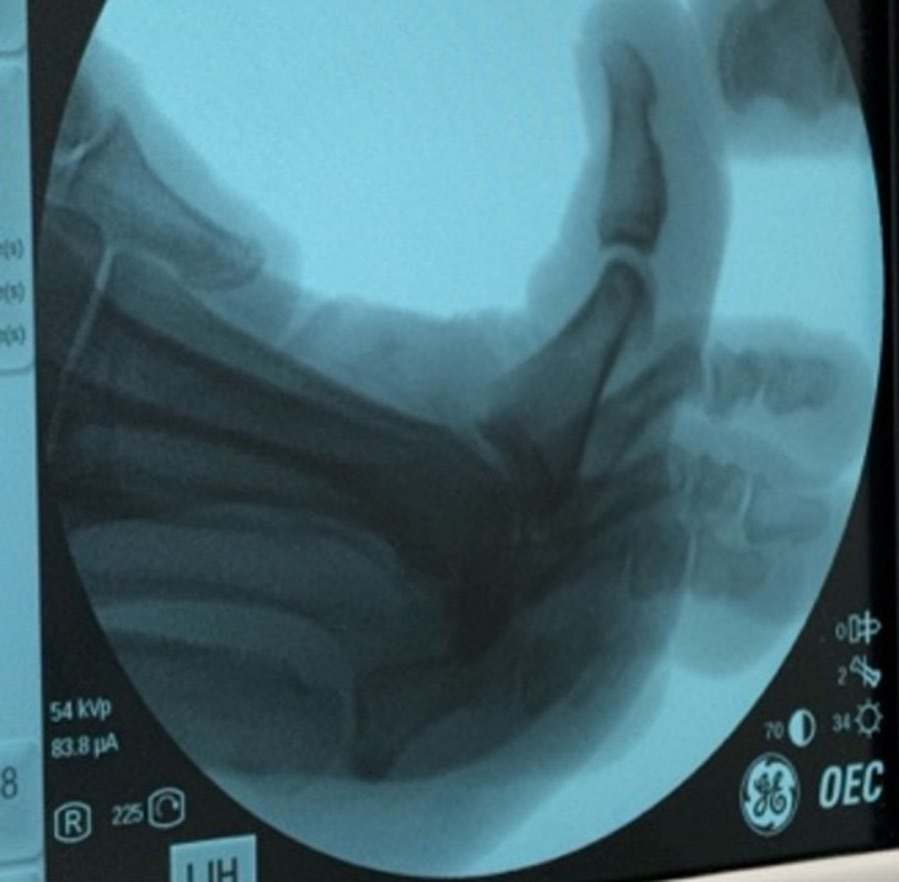
Intraoperative fluoroscopic images (mini C-arm) of the first ray after cheilectomy. The panel demonstrates the intraoperative assessment of dorsiflexion of the hallux following resection, confirming improved range of motion.

**Figure 5.**
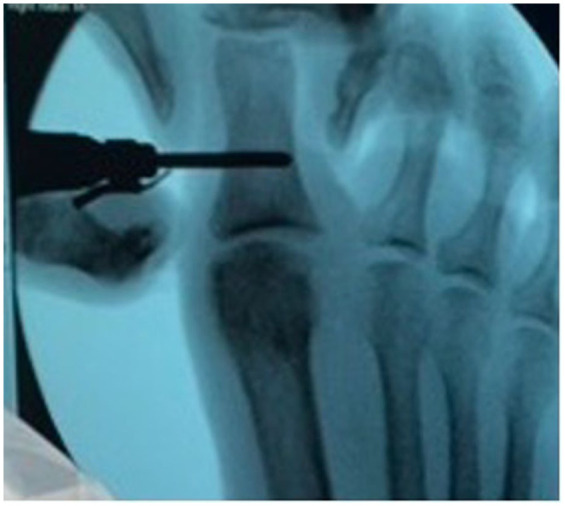
Intraoperative fluoroscopic image (mini C-arm) showing dorsal closing wedge resection. A 2 × 12-mm Shannon burr is shown advanced perpendicularly into the proximal phalanx to perform a dorsal closing wedge resection as part of the Moberg osteotomy.

**Figure 6.**
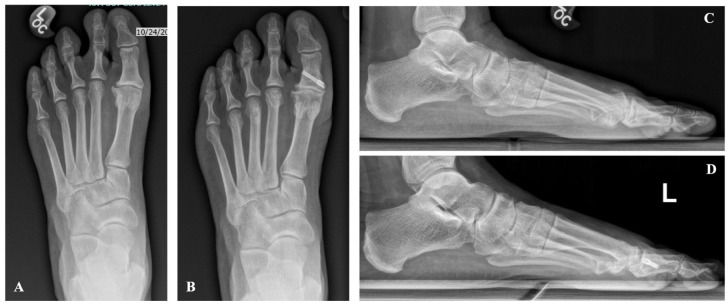
Weightbearing radiographs of the left foot. (A) Preoperative anteroposterior view. (B) Six-month postoperative anteroposterior view. (C) Preoperative lateral view. (D) Six-month postoperative lateral view. Images demonstrate radiographic outcomes following Moberg osteotomy and cheilectomy.

After performing a popliteal nerve block, a 2-mm stab incision was made just medial to the extensor hallucis longus about 2 cm proximal to the MTP (Figure 2). A soft tissue elevator was advanced to elevate the soft tissues from the dorsal bone spur. Next, a 3-mm wedge burr was advanced into the wound to perform an outside-in resection of the spur, aiming at removing about 20% of the metatarsal head (Figure 3). On completion of the cheilectomy bone debris was removed using a rasp as well as copious irrigation using an 18-gauge blunt needle (Figure 4). Next, a stab incision was made at the medial aspect of the proximal phalanx base, about 1 cm distal to the MTP. A 2 × 12-mm Shannon burr was advanced perpendicularly into the bone to complete a dorsal closing wedge resection. Next, the burr was exchanged for a 3-mm wedge burr to further open up the osteotomy with a single pass (Figure 5). The osteotomy was manually reduced and stabilized with a 3-mm headless screw (Figure 6).

#### Postoperative management

Postoperative medications included ibuprofen 600 mg 3 times per day for the first 3 days, as well as 20 tablets of 5 mg oxycodone to be taken as needed for pain. Patients were asked to log the number of oxycodone tablets taken in the first 14 days after the procedure.

No specific physical therapy or home exercise program was prescribed in the postoperative period.

#### Statistical analysis

No a priori power analysis was performed to determine the sample size. The study sample was based on the number of eligible patients treated during the study period.

All statistical analyses were performed using independent 2-sample *t* tests to compare continuous variables between 2 groups. Welch *t* test was applied to account for possible unequal variances between groups. Group sizes were n = 43 and n = 53, respectively. Data were expressed as mean ± SD. A 2-tailed *P* value less than .05 was considered statistically significant.

## Results

A total of 96 patients were included in the study, of whom 43 underwent open Moberg osteotomy and 53 underwent the percutaneous technique. All procedures were performed in combination with dorsal cheilectomy.

Baseline demographic characteristics, including age, sex distribution, and follow-up duration, were comparable between the 2 groups, with no significant differences observed as displayed in Table 1.

**Table 1.** Patient Demographics.

Variable	Open Group (n = 43)	Percutaneous Group (n = 53)	*P* Value
Mean age, y (SD)	67.2 (9.5)	67.1 (9.0)	.94
Age range, y	32-88	48-86	–
Sex, n (%)			.556
Male	7 (16.3)	6 (11.3)	
Female	36 (83.7)	47 (88.7)	
Follow-up, mo (SD)	31.2 (3.7)	33.2 (5.1)	.029
Follow-up range, mo	25-38	24-42	–

The mean preoperative VAS score was 7.40 (SD 0.88, range 6-9) in the open group and 7.40 (SD 0.86, range 6-9) in the percutaneous group. At final follow-up, the mean VAS decreased to 0.19 (SD 0.39, range 0-1) in the open group and 0.32 (SD 0.67, range 0-4) in the percutaneous group.

The reduction in pain was statistically significant in both groups (*P* < .0001 for both). The mean improvement (ΔVAS) was −7.21 in the open group and −7.08 in the percutaneous group. There was no statistically significant difference in pain reduction between the 2 techniques (*P* = .525).

The FFI showed a significant improvement in all domains for both the open and percutaneous techniques.

For the FFI pain subscale, the open group improved from a mean of 21.26 (SD 3.51) to 4.58 (SD 2.08), *P* < .0001, and the percutaneous group from 20.96 (SD 3.13) to 4.47 (SD 1.83), *P* < .0001. The comparison of postoperative scores between the 2 groups did not show a statistically significant difference (*P* = .69).

For the FFI disability subscale, scores in the open group decreased from 16.65 (SD 2.80) to 5.56 (SD 2.07), *P* < .0001, and from 16.91 (SD 3.11) to 5.15 (SD 2.00) in the percutaneous group, *P* < .0001. No significant difference was found between the postoperative scores of the 2 groups (*P* = .31).

For the FFI activity limitation subscale, the open group showed a reduction from 18.58 (SD 2.18) to 6.84 (SD 2.84), *P* < .0001, whereas the percutaneous group improved from 18.32 (SD 2.45) to 6.87 (SD 3.06), *P* < .0001. The postoperative comparison showed no significant difference (*P* = .95).

Finally, the total FFI score dropped significantly from 56.67 (SD 4.73) to 16.98 (SD 4.91) in the open group, *P* < .0001, and from 56.28 (SD 5.30) to 16.49 (SD 5.29) in the percutaneous group, *P* < .0001. Postoperative total scores were comparable between groups (*P* = .59) (Table 2).

**Table 2.** VAS and FFI Results (Open vs Percutaneous).

Outcome	Group	Preop. Mean (SD)	Postop. Mean (SD)	Range Postop.	Pre- vs Postop. *P*	Postop. Between-Group *P*
VAS	Open	7.40 (0.88)	0.19 (0.39)	0-1	<.0001	.15
	Percutaneous	7.40 (0.86)	0.32 (0.67)	0-4	<.0001	
FFI pain	Open	21.26 (3.51)	4.58 (2.08)	2-9	<.0001	.69
	Percutaneous	20.96 (3.13)	4.47 (1.83)	2-9	<.0001	
FFI disability	Open	16.65 (2.80)	5.56 (2.07)	3-10	<.0001	.31
	Percutaneous	16.91 (3.11)	5.15 (2.00)	2-10	<.0001	
FFI activity	Open	18.58 (2.18)	6.84 (2.84)	3-12	<.0001	.95
	Percutaneous	18.32 (2.45)	6.87 (3.06)	3-13	<.0001	
FFI total	Open	56.67 (4.73)	16.98 (4.91)	8-31	<.0001	.59
	Percutaneous	56.28 (5.30)	16.49 (5.29)	9-30	<.0001	

Abbreviations: FFI, Foot Function Index; VAS, visual analog scale.

With 43 patients in the open group and 53 in the minimally invasive group (2-sided α = 0.05), a difference of 2 points on the VAS (SD = 2, Cohen *d* = 1.0) yields an estimated power of 99.8%. For the FFI total score, a difference of 10 points (SD = 15, Cohen *d*≈0.67) corresponds to an estimated power of 90.1%. Thus, the available sample sizes are sufficient to reliably detect clinically meaningful improvements on both VAS and FFI.

The number of oxycodone pills consumed during the first 2 postoperative weeks was recorded for both groups. In the open group, the mean number of pills taken was 13.3, with an SD of 6.1 and a range from 6 to 25. In the percutaneous group, the mean number of pills taken was 3.6, with an SD of 1.9 and a range from 1 to 10. The *P* value for this comparison was <.0001, showing a statistically significant inferior use of oxycodone pills in the percutaneous group. Post hoc power analysis demonstrated that the study was adequately powered to detect the large observed difference in postoperative opioid consumption.

In the open group (n = 43), 2 cases of wound dehiscence occurred (4.7%), both managed conservatively. In the percutaneous group (n = 53), no wound complications were observed. The comparison of wound complications between the open group (2/43) and the percutaneous group (0/53) did not reach statistical significance (*P* = .20).

In the percutaneous group, 1 patient (2.3%) required conversion to first metatarsophalangeal joint arthrodesis 12 months after surgery because of persistent symptoms. In the open group, 1 patient (1.9%) underwent arthrodesis 6 months postoperatively for persistent pain. Reoperation rates were comparable between groups (open 2.3% [1/43] vs percutaneous 1.9% [1/53], *P* > .99) (Table 3).

**Table 3.** Complications and Reoperation Rate.

Complication	Open Group, n (%) (n = 43)	Percutaneous Group, n (%) (n = 53)	*P* Value
Wound dehiscence	2 (4.7)	0 (0)	.20
Reoperation (arthrodesis)	1 (2.3)	1 (1.9)	.10

We did not observe any MIS-specific complications such as nerve injury/neuritis or skin/bone thermal injury in either group. Likewise, no cases of delayed union or malunion were detected in either group.

## Discussion

This study compared the effectiveness and safety of percutaneous Moberg osteotomy combined with percutaneous dorsal cheilectomy vs the traditional open technique for the treatment of hallux rigidus. The findings demonstrate that both surgical approaches lead to significant improvements in pain relief and functional outcomes, with no clinically meaningful differences between the 2 groups at a minimum follow-up of 2 years, providing short to midterm clinical outcomes. Because the open cohort (2016-2018) and the percutaneous cohort (2018-2022) were treated in different eras, changes in perioperative care, including opioid-prescribing norms, may partially explain the lower opioid use observed with the percutaneous approach.

Both techniques resulted in a marked reduction in pain, with very low postoperative VAS scores, indicating excellent analgesic efficacy regardless of the surgical approach. Similarly, the FFI showed substantial and statistically significant improvement across all domains—pain, disability, and activity limitation—in both the open and percutaneous groups without significant differences.

One particularly noteworthy finding was the significantly lower consumption of oxycodone in the percutaneous group during the first 2 postoperative weeks. This suggests a potential advantage of the percutaneous approach in terms of postoperative pain management and highlights its role in reducing opioid use, a particularly relevant consideration in contemporary clinical practice.

Cheilectomy is a widely used and well-established surgical technique for the treatment of hallux rigidus grade I or II, with consistently favorable outcomes reported in the literature in terms of pain relief, functional improvement, and motion preservation.^18-20^ When combined with Moberg osteotomy, several advantages arise, as it aims to shift the load to the plantar aspect of the foot, thereby compensating for the limited dorsiflexion and improving the range of motion.^21,22^

The benefits of percutaneous cheilectomy are documented in the literature, particularly with regard to reduced soft tissue trauma, faster recovery, and decreased postoperative pain.^23-25^

However, there is a notable lack of studies focusing on the percutaneous Moberg osteotomy. De Arruda et al^26^ assessed a small cohort of 7 patients undergoing percutaneous cheilectomy combined with Watermann and Moberg osteotomies. They reported significant improvements in pain (VAS), function (American Orthopaedic Foot & Ankle Society score), and dorsiflexion, with no postoperative complications. Although their results are promising, the small sample size, short follow-up (mean 8 months), and lack of a control group limit the generalizability of their findings. In contrast, our study confirms similar benefits—particularly in terms of pain relief and functional improvement—within a much larger cohort and with longer follow-up, offering more robust evidence of the technique’s effectiveness.

DiGiovanni et al^15^ compared open cheilectomy with Moberg osteotomy to percutaneous cheilectomy alone. They observed a modestly greater improvement in Patient-Reported Outcome Measures Information System (PROMIS) scores in the open group but found no difference in reoperation rates. Importantly, in their study the Moberg osteotomy was performed exclusively through an open approach; the percutaneous group underwent cheilectomy alone, without any osteotomy. Therefore, their findings do not provide information on the outcomes of a percutaneous Moberg osteotomy, which remains an area scarcely explored in the literature.

Our results fill this critical gap, demonstrating that adding a percutaneous Moberg osteotomy to the cheilectomy allows the fully percutaneous approach to match the outcomes of the open procedure in terms of pain (VAS), functional scores (FFI), and complication rates—while also reducing early postoperative opioid use. These findings strongly support the percutaneous Moberg + cheilectomy as a safe and effective alternative to the traditional open method, with added advantages in early postoperative recovery.

To our knowledge, it is the first published study specifically evaluating percutaneous Moberg osteotomy in combination with dorsal cheilectomy This retrospective, nonrandomized study is subject to selection and measurement bias, and the 2 cohorts were treated in different time periods (open 2016-2018 vs percutaneous 2018-2022), introducing potential era effects on perioperative care and opioid prescribing. Small numbers for some complications limit power; therefore, nonsignificant differences (eg, wound events) should be interpreted cautiously. Outcomes such as opioid consumption were self-reported and may be influenced by prescription size, counseling, and social factors not captured here. We did not perform adjusted analyses; future studies should use multivariable or matched designs to address confounding. Longer-term durability beyond 2-3 years remains unknown.

Despite these limitations, this study provides encouraging evidence that percutaneous Moberg osteotomy combined with dorsal cheilectomy may be a safe and effective alternative to the open technique. It offers comparable functional outcomes with the added benefits of reduced opioid consumption. These findings support the integration of minimally invasive approaches in the surgical management of hallux rigidus.

## Conclusions

In our study, we found percutaneous that Moberg osteotomy combined with percutaneous dorsal cheilectomy represents a safe and effective surgical option for the treatment of hallux rigidus. In this comparative study, the percutaneous approach demonstrated equivalent outcomes to the open technique in terms of pain relief and functional improvement, with lower early opioid consumption observed in the percutaneous cohort. Given the nonconcurrent groups and retrospective design, this association should be confirmed in prospective studies.

## Supplemental Material

sj-pdf-1-fao-10.1177_24730114251388095 – Supplemental material for Open vs Percutaneous Moberg Osteotomy With Dorsal Cheilectomy for Hallux Rigidus: A Retrospective Comparative Study of Surgical Approaches With Minimum 2 Years’ Follow-up Supplemental material, sj-pdf-1-fao-10.1177_24730114251388095 for Open vs Percutaneous Moberg Osteotomy With Dorsal Cheilectomy for Hallux Rigidus: A Retrospective Comparative Study of Surgical Approaches With Minimum 2 Years’ Follow-up by Alice Montagna, Paolo Ivan Fiore, Enrico Pozzessere and Ettore Vulcano in Foot & Ankle Orthopaedics
